# Supplementary material for: Reporter Gene-Based qRT-PCR Assay for Rho-Dependent Termination In Vivo
Source: Cells. 2023 Nov 9;12(22):2596. doi: 10.3390/cells12222596 (PMC10670590; doi:10.3390/cells12222596)
Supplement: Supplementary file 1 [file cells-12-02596-s001.zip › cells-2639780-supplementary.pdf]

SUPPLEMENTAL FIGURE

Figure S1.

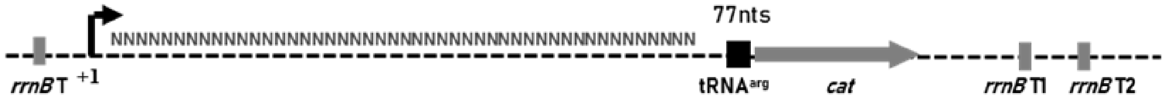

TTCCCAGGCATCAAATAAAACGAAAGGCTCAGTCGAAAGACTGGGCCTTTCGTTTTATCTGTTGTTTGTCGGTG *rrnB* T
AACGCTCTCCTGAGTAGGACAAATCCGCCGGGAGCGGATTTGAACGTTGCGAAGCAACGGCCCGGAGGGTGG
CGGGCAGGACGCCCGCCATAAACTGCCAGGGAATTCCCGGGGATCCNNNNNNNNNNNNNNNNNNN ***gal* sequence**
NNNNNNNNNNNNNNNNNNNNNNNNNNNNNNNNNNNNNNNNNNNNNNNNNNNNNNNNNNNNNN
NNNNNNNNNNNGTCGACCTGCAGCCAAGCTTGCGCTCGTAGCTCAGTTGGATAGAGTACA tRNA^{arg} (*argX*)
GGTTTCCGAAGCCTGGGGTCGTGGGTTCGATCCCCGCCGGGCGCACCAGAGTAGGACAAAT
CCGCCGAGCTTCGACGAGATTTTCAGGAGCTAAGGAAGCTAAAATGGAGAAAAAAATCACTGGATATACCAC
CGTTGATATATCCCAATCGCATCGTAAAGAACATTTTGAGGCATTTCAGTCAGTTGCTCAATGTACCTATAACC
AGACCGTTCAGCTGGATATTACGGCCTTTTTAAAGACCGTAAAGAAAAATAAGCACAAGTTTTATCCGGCCTT
TATTCACATTCTTGCCCGCCTGATGAATGCTCATCCGGAATTCCGTATGGCAATGAAAGACGGTGAGCTGGTG
ATATGGGATAGTGTTCACCCTTGTTACACCGTTTTCCATGAGCAAACTGAAACGTTTTCATCGCTCTGGAGTGA *cat*
ATACCACGACGATTTCCGGCAGTTTCTACACATATATTCGCAAGATGTGGCGTGTTACGGTGAAAACCTGGCC
TATTTCCCTAAAGGGTTTATTGAGAATATGTTTTTCGTCTCAGCCAATCCCTGGGTGAGTTTCACCAGTTTTGAT
TTAAACGTGGCCAATATGGACAACTTCTTCGCCCCCGTTTTCACCATGGGCAAATATTATACGCAAGGCGACA
AGGTGCTGATGCCGCTGGCGATTCAGGTTCATCATGCCGTCTGTGATGGCTTCCATGTCGGCAGAATGCTTAAT
GAATTACAACAGTACTGCGATGAGTGGCAGGGCGGGGCGTAATTTTTTTAAGGCAGTTATTGGTGCCCTTAAA
CGCCTGGTGCTACGCCTGAATAAGTGATAATAAGCGGATGAATGGCAGAAATTCGTCGAGGCGGCACCTCGCT
AACGGATTCACCACTCCAAGAATTGGAGCCAATCAATTCTTGCGGAGAACTGTGAATGCGCAAACCAACCCTT
GGCAGAACATATCCATCGCGTCCGCCATCTCCAGCAGCCGCACGCGGCGCATCTCGGCTGTTTTGGCGGATGA
GAGAAGATTTTCAGCCTGATACAGATTAAATCAGAACGCAGAAGCGGTCTGATAAAACAGAATTTGCCTGGC
GGCAGTAGCGCGGTGGTCCCACCTGACCCCATGCCGAACTCAGAAGTGAAACGCCGTAGCGCCGATGGTAGT
GTGGGGTCTCCCCATGCGAGAGTAGGGAACTGCCAGGCATCAAATAAAACGAAAGGCTCAGTCGAAAGACTG *rrnB* T
GGCCTTTCGTTTTATCTGTTGTTTGTCGGTGAACGCTCTCCTGAGTAGGACAAATCCGCCGGGAGCGGATTTGA
ACGTTGCGAAGCAACGGCCCGGAGGGTGGCGGGCAGGACGCCCGCCATAAACTGCCAGGCATCAAATTAAGC
AGAAGGCCATCCTGACGGATGGCCTTTTTGCGTTTCTACAAACTCTTCCTGTCGTCATATCTACAAGCCATCCC
CCCACAGATACGGTAAACTAGCCTCGTTTTTGCATCAGGAAAGCAGCTGTTTTGGCGGATGAGAGAAGATTTT
CAGCCTGATACAGATTAAATCAGAACGCAGAAGCGGTCTGATAAAACAGAATTTGCCTGGCGGCAGTAGCGC
GGTGGTCCCACCTGACCCCATGCCGAACTCAGAAGTGAAACGCCGTAGCGCCGATGGTAGTGTGGGGTCTCCC
CATGCGAGAGTAGGGAACTGCCAGGCATCAAATAAAACGAAAGGCTCAGTCGAAAGACTGGGCCTTTCGTTT *rrnB* T
TATCTGTTGTTTGTCGGTGAACGCTCTCCTGAGTAGGACAAATCCGCCGGGAGCGGATTTGAACGTTGCGAAG
CAACGGCCCGGAGGGTGGCGGGCAGGACGCCCGCCATAAACTGCCAGGCATCAAATTAAGCAGAAGGCCATC
CTGACGGATGGCCTTTTTGCGTTTCTACAAACTCTTCCTGTCGTCATATCTACAAGCCATCCCCCCACAGATAC
GGTAAACTAGCCTCGTTTTTGCATCAGGAAAGCAGTCGGGCAGCGTTGGGTCCTGGCCACGGGTGCGCATGAT
CGTGCTCCTGTCGTTGAGGACCCGGCTAGGCTGGCGGGGTTGCCTTACTGGTTAGCAGAATGAATCACCGATA

CGCGAGCGAACGTGAAGCGACTGCTGCTGCAAAACGTCTGCGACCTGAGCAACAACATGAATGGTCTTCGGTT
TCCGTGTTTCGTAAAGTCTGGAAACGCGGAAGTCAGCGCCCTGCACCATTATGTTCCGGATCTGCATCGCAGG
ATGCTGCTGGCTACCCTGTGGAACACCTACATCTGTATTAACGAAGCGCTGGCATTGACCCTGAGTGATTTTTC
TCTGGTCCCGCCGCATCCATACCGCCAGTTGTTTACCCTCACAACGTTCCAGTAACCGGGCATGTTCATCATCA
GTAACCCGTATCGTGAGCATCCTCTCTCGTTTCATCGGTATCATTACCCCCATGAACAGAAATCCCCCTTACAC
GGAGGCATCAGTGACCAAACAGGAAAAAACCGCCCTTAACATGGCCCGCTTTATCAGAAGCCAGACATTAAC
GCTTCTGGAGAAACTCAACGAGCTGGACGCGGATGAACAGGCAGACATCTGTGAATCGCTTCACGACCACGC
TGATGAGCTTTACCGCAGCTGCCTCGCGCGTTTCGGTGATGACGGTGAAAACCTCTGACACATGCAGCTCCCG
GAGACGGTCACAGCTTGTCTGTAAGCGGATGCCGGGAGCAGACAAGCCCGTCAGGGCGCGTCAGCGGGTGTT
GGCGGGTGTCGGGGCGCAGCCATGACCCAGTCACGTAGCGATAGCGGAGTGTATACTGGCTTAACTATGCGG
CATCAGAGCAGATTGTACTGAGAGTGCACCATATGCGGTGTGAAATACCGCACAGATGCGTAAGGAGAAAAT
ACCGCATCAGGCGCTCTTCCGCTTCCTCGCTCACTGACTCGCTGCGCTCGGTCGTTCGGCTGCGGCGAGCGGTA
TCAGCTCACTCAAAGGCGGTAATACGGTTATCCACAGAATCAGGGGATAACGCAGGAAAGAACATGTGAGCA
AAAGGCCAGCAAAAGGCCAGGAACCGTAAAAAGGCCGCGTTGCTGGCGTTTTTCCATAGGCTCCGCCCCCCTG
ACGAGCATCACAAAAATCGACGCTCAAGTCAGAGGTGGCGAAACCCGACAGGACTATAAAGATACCAGGCGT
TTCCCCCTGGAAGCTCCCTCGTGCGCTCTCCTGTTCCGACCCTGCCGCTTACCGGATACCTGTCCGCCTTTCTCC
CTTCGGGAAGCGTGGCGCTTTCTCAATGCTCACGCTGTAGGTATCTCAGTTCGGTGTAGGTCGTTCGCTCCAAG
CTGGGCTGTGTGCACGAACCCCCCGTTCAGCCCGACCGCTGCGCCTTATCCGGTAACTATCGTCTTGAGTCCAA
CCCGGTAAGACACGACTTATCGCCACTGGCAGCAGCCACTGGTAACAGGATTAGCAGAGCGAGGTATGTAGG
CGGTGCTACAGAGTTCTTGAAGTGGTGGCCTAACTACGGCTACACTAGAAGGACAGTATTTGGTATCTGCGCT
CTGCTGAAGCCAGTTACCTTCGGAAAAAGAGTTGGTAGCTCTTGATCCGGCAAACAAACCACCGCTGGTAGCG
GTGGTTTTTTTGTTTGCAAGCAGCAGATTACGCGCAGAAAAAAAGGATCTCAAGAAGATCCTTTGATCTTTTCT
ACGGGGTCTGACGCTCAGTGGAACGAAAACTCACGTTAAGGGATTTTGGTCATGAGATTATCAAAAAGGATCT
TCACCTAGATCCTTTTAAATTAAAAATGAAGTTTTAAATCAATCTAAAGTATATATGAGTAAACTTGGTCTGAC
AGTTACCAATGCTTAATCAGTGAGGCACCTATCTCAGCGATCTGTCTATTTCGTTCATCCATAGTTGCCTGACT
CCCCGTCGTGTAGATAACTACGATACGGGAGGGCTTACCATCTGGCCCCAGTGCTGCAATGATACCGCGAGAC
CCACGCTCACCGGCTCCAGATTTATCAGCAATAAACCAGCCAGCCGGAAGGGCCGAGCGCAGAAGTGGTCCT
GCAACTTTATCCGCCTCCATCCAGTCTATTAATTGTTGCCGGGAAGCTAGAGTAAGTAGTTCGCCAGTTAATAG
TTTGCGCAACGTTGTTGCCATTGCTGCAGGCATCGTGGTGTCACGCTCGTCGTTTGGTATGGCTTCATTCAGCT
CCGGTTCCCAACGATCAAGGCGAGTTACATGATCCCCCATGTTGTGCAAAAAAGCGGTTAGCTCCTTCGGTCC
TCCGATCGTTGTCAGAAGTAAGTTGGCCGCAGTGTTATCACTCATGGTTATGGCAGCACTGCATAATTCTCTTA
CTGTCATGCCATCCGTAAGATGCTTTTCTGTGACTGGTGAGTACTCAACCAAGTCATTCTGAGAATAGTGTATG
CGGCGACCGAGTTGCTCTTGCCCGGCGTCAACACGGGATAATACCGCGCCACATAGCAGAACTTTAAAAGTGC
TCATCATTGGAAAACGTTCTTCGGGGCGAAAACTCTCAAGGATCTTACCGCTGTTGAGATCCAGTTCGATGTA
ACCCACTCGTGCACCCAACTGATCTTCAGCATCTTTTACTTTCACCAGCGTTTCTGGGTGAGCAAAAACAGGAA
GGCAAAATGCCGCAAAAAAGGGAATAAGGGCGACACGGAAATGTTGAATACTCATACTCTTCCTTTTTCAATA
TTATTGAAGCATTTATCAGGGTTATTGTCTCATGAGCGGATACATATTTGAATGTATTTAGAAAAATAAACAA
ATAGGGGTTCCGCGCACATTTCCCCGAAAAGTGCCACCTGACGTCTAAGAAACCATTATTATCATGACATTAA
CCTATAAAAATAGGCGTATCACGAGGCCCTTTCGTCTTCAAGAA

Figure S1. Schematic presentation of pKK232-8-derived plasmids.

Top: A schematic description of a plasmid that was used to assess Rho-dependent transcription

termination (RDT). The plasmid contains the *rrnB* transcription terminator located ahead of the *gal* sequences that are to be inserted (N) to stop abnormal transcription from the plasmid DNA with transcriptionally fused *argX* gene (77 bp long) encoding the $tRNA^{arg}$ from *B. albidum*, and the *cat* gene, which is fused to the 3' end of the $tRNA^{arg}$, followed by the *rrnB* transcription terminators.

Bottom: Sequence presentation of functional regions of a plasmid that was used to assess RDT. The region with the inserted *gal* (denoted as N) and $tRNA^{arg}$ sequences are highlighted in purple and turquoise, respectively. The *gal* sequences (purple) are inserted between *Bam*HI and *Sal*I. Recognition sites of restriction enzymes *Bam*HI and *Sal*I are underlined. The chloramphenicol resistance (*cat*) gene and the strong Rho-independent *rrnB* transcription termination signals are highlighted in gray and green. The qRT-PCR primers for $tRNA^{arg}$ quantitation are highlighted in red double-underline.

SUPPLEMENTAL TABLE

Table S1. Primers used in this study.

**Primer name**	**Primer sequences (5′→3′)**	**Usage**
rrsB-F	CAGAATGCCACGGTGAATACGTT	qRT-PCR
rrsB-R	CAACCCAACTCCCATGGTGTGA	
$tRNA^{arg}$-F	GCTCAGTTGGATAGAGTACA	qRT-PCR for
$tRNA^{arg}$-R	TGGTGCGCCCGGCGGGGA	detecting $tRNA^{arg}$
gal+70-F	CTATAGGGCGGATCCAATTCTTGTGTA	
gal+1031-R	ACGCGTCGACTCCCTGTGGATGGCGTGACT	
gal+1193-R	GCAATCTGGATCGTGCGCAGGTAAC	
gal+2000-R	GTCTCTGCCAGGTCGACATAACCAACCA	$tRNA^{arg}$ plasmid
gal+2184-R	CCAATCAAATTGTCGACGCCAGGCGCCT	construction for
gal+4270-R	ACATTACTCAGGTCGACACTGATATTCC	qRT-PCR
gal+4344-R	TGAGTTGCAAAGTCGACGGTGTTTGCTG	
gal+4444-R	TGATAATAATTGTCGACATATTGCCGCG	
